# Maternal serum proteomic profiles of pregnant women with type 1 diabetes

**DOI:** 10.1038/s41598-022-12221-5

**Published:** 2022-05-24

**Authors:** Paweł Gutaj, Jan Matysiak, Eliza Matuszewska, Katarzyna Jaskiewicz, Dorota Kamińska, Agata Światły-Błaszkiewicz, Tomasz Szczapa, Anastasia Kalantarova, Marzena Gajecka, Ewa Wender-Ozegowska

**Affiliations:** 1 Department of Reproduction, Poznan University of Medical Sciences, 61-701 Poznan, Poland; 2 Department of Inorganic and Analytical Chemistry, Poznan University of Medical Sciences, 61-701 Poznan, Poland; 3 Chair and Department of Genetics and Pharmaceutical Microbiology, Poznan University of Medical Sciences, 61-701 Poznan, Poland; 4 Institute of Human Genetics, Polish Academy of Sciences, 60-479 Poznan, Poland; 5 Department of Inorganic and Analytical Chemistry, Faculty of Pharmacy, Collegium Medicum in Bydgoszcz, Nicolaus Copernicus University in Toruń, Jurasza 2, 85-089 Bydgoszcz, Poland; 6 Department of Neonatology, Neonatal Biophysical Monitoring and Cardiopulmonary Therapies Research Unit, Poznan University of Medical Sciences, 61-701 Poznan, Poland; 7 Medicine Program, Poznan University of Medical Sciences, 61-701 Poznan, Poland

**Keywords:** Medical research, Biomarkers

## Abstract

Despite improvement in the care of diabetes over the years, pregnancy complicated by type 1 diabetes (T1DM) is still associated with adverse maternal and neonatal outcomes. To date, proteomics studies have been conducted to identify T1DM biomarkers in non-pregnant women, however, no studies included T1DM pregnant women. In this study serum proteomic profiling was conducted in pregnant women with T1DM in the late third trimester. Serum samples were collected from 40 women with T1DM and 38 healthy controls within 3 days before delivery at term pregnancy. Significant differences between serum proteomic patterns were revealed, showing discriminative peaks for complement C3 and C4-A, kininogen-1, and fibrinogen alpha chain. Quantification of selected discriminative proteins by ELISA kits was also performed. The serum concentration of kininogen-1 was significantly lower in women with T1DM than in controls. There were no significant differences in serum concentrations of complement C3 and complement C4-A between study groups. These data indicate that pregnant women with T1DM have a distinct proteomic profile involving proteins in the coagulation and inflammatory pathways. However, their utility as biomarkers of pregnancy complications in women with T1DM warrants further investigation.

## Introduction

The incidence of type 1 diabetes mellitus (T1DM) is rising worldwide, reaching 15 per 100,000. It is a disease that typically has an onset in childhood or early adulthood^1^. Therefore, the number of women with T1DM entering reproductive age is expected to grow. Many studies have been conducted on the pathophysiology of diabetes in the pregnant state. It is now clear that T1DM influences both maternal and fetal/neonatal health^2^. In women with T1DM, pregnancy can exacerbate preexisting conditions, such as retinopathy^3^. Moreover, these women are at exceptionally high risk of developing preeclampsia which is now considered one of the most significant factors influencing maternal and fetal mortality rates in developed countries^4^. Another spectrum of diabetes-related complication is fetal growth abnormalities. Depending on patients' characteristics, diabetes can promote both large for gestational age (LGA) and fetal growth restriction (FGR). The first complication is observed mainly in poorly controlled women without vasculopathy. Such factors as maternal lipids, gestational weight gain, and alterations in adipokines have also been identified as promotors of LGA^5,6^. FGR is typically observed in women with long-lasting diabetes accompanied by vasculopathy^7^. However, the occurrence of many diabetes complications is impossible to explain by simple clinical factors. The rapid development of high- throughput omics technologies facilitates the search for novel biomarkers of T1DM that may be involved in the development of the disease^8^. These biomarkers can be useful in the prediction and early diagnosis of the disease and its complications, such as retinopathy and nephropathy in the non-pregnant state^9,10^. More specifically, a recent study suggested that specific proteins (ie. alpha-2-macroglobulin, β2 glycoprotein I and Ig alpha-2 chain C region) are potential biomarkers of diabetic retinopathy and nephropathy in non-pregnant patients with T1DM^8^. While another study used proteomic technology to further analyze the mechanism by which kidney injury in diabetes occurs, concluding that there is a sequence of events and key proteins at each stage involved in the development of this complication^9^. Collectively this information from proteomic studies provides a better clinical picture of T1DM complications and provides a potential for screening patients. In a similar vein, proteomics technologies give a new insight into biological processes involved in several clinical problems associated with pregnancy, including pre-term birth, preeclampsia, and fetal growth alterations^11^.

Since, to the authors' knowledge, such studies have not yet been conducted in women with T1DM,the aim of this project was to establish specific serum proteomic profile in pregnant women with T1DM, characterise it, and finally correlate the results with chosen clinical and laboratory parameters. Discovery of a unique proteomic profile in patients with T1DM will lay the ground work for future studies to unravel how such markers can be used to clinical practice to establish the diagnosis or monitor for the risk to develop complications.

## Materials and methods

### Participants

A study of 40 pregnant women with T1DM and 38 healthy controls was conducted in the Department of Reproduction in Gynecologic and Obstetrical University Hospital in Poznan, Poland. The Department, including its outpatient clinic, is the biggest perinatal center for pregnant women with diabetes in Poland. It provides care for patients from the Greater Poland voivodeship, with a population of approximately 3.4 million. Inclusion criteria for both the study and control groups included age (18–45 years) and term pregnancy (37 + 0–41 + 0 weeks of gestation) with well controlled T1DM. The process of care delivered to T1DM women without complications was based on at least 3 planned, short-stay hospital admissions during pregnancy: in the first trimester, in mid-pregnancy (20–24 weeks of gestation), and near delivery (34–39 weeks of gestation). Patients who required more vigilant surveillance were admitted more frequently. In-between hospital admissions, patients were referred biweekly for regular check-ups in the hospital-based outpatient clinic and also received regular outpatient consultations with a specialist in diabetology. Hospitalized T1DM patients also had regular consultations with certified Diabetes Nurse Educators focusing on proper insulin therapy, glycemic control, and dietary education. The Control group consisted of consecutive, unselected, healthy pregnant women admitted to the clinic at term pregnancy.

Because the current study was an arm of a larger project on maternal and neonatal microbiomes, we established the following exclusion criteria for both women with T1DM and controls: pre-term delivery, multiple pregnancies, genitourinary infections diagnosed in the last 4 weeks, use of oral or vaginal antibiotics/antifungal medicines/probiotic supplements in pregnancy in the previous 4 weeks; vaginal irrigation/sexual intercourse in last 72 h.

All women underwent detailed nutritional assessment, using 24-h dietary recall for 7 days. Moreover, information about the participants and their dietary habits was collected based on the author's survey and the assessment of the validated Food Frequency Questionnaire FFQ-D10. Detailed protocols of nutritional assessment and dietary habits, as well as its results, were discussed in detail elsewhere^12^.

### Sample pretreatment

In this study, we examined human serum samples derived from ascertained women. The blood samples from each patient were collected into 9 mL sterile S-Monovette tubes (SARSTED AG & Co. KG, Numbrecht, Germany) with a clotting activator present. The blood was left to clot for 30 min at room temperature, and then within 3 h from collection time, centrifuged at 2000*g* for 10 min at room temperature (RT). The serum supernatant was carefully removed and again centrifuged at 2000*g* for 10 min at RT. After last centrifugation, the serum supernatant was collected, aliquoted, and frozen at − 80 °C. Before mass spectrometry analyses, all biological samples were concentrated, desalted, and purified using ZipTip C18 (Millipore, Bedford, MA, USA) reverse phase chromatography micropipette tips, according to the manufacturer's protocol. This step included dilution the samples with 0.1% trifluoroacetic acid (TFA) in water (in ratio 1:5), loading such mixtures onto ZipTip tips, and eluting the adsorbed proteins and peptides with 50% ACN in 0.1% TFA. For the tips conditioning, acetonitrile (ACN) and 0.1% TFA in water were used.

### MALDI-TOF MS protein-peptide profiling

Prior to MALDI-TOF MS (matrix-assisted laser desorption/ionisation-time of flight mass spectrometry) analyses, ZipTip C18 eluents were mixed separately with matrix solution in a 1:10 ratio. The matrix solution consisted of 0.3 g/L α-cyano-4-hydroxycinnamic acid (HCCA) in a 2:1 mixture of ethanol/acetone (v/v). The eluent-matrix mixtures were spotted manually in triplicates onto the AnchorChip Standard (Bruker Daltonics, Bremen, Germany) target plate. UltrafleXtreme (Bruker Daltonics, Bremen, Germany) MALDI-TOF mass spectrometer was used for MS analysis. All the experiments were conducted in a linear-positive mode, and ions were analysed in the m/z range of 1000–10,000. Each spectrum was interrogated using 2000 laser shots. External calibration was performed with a mixture of Protein Calibration Standard I and Peptide Calibration Standard (Bruker Daltonics, Bremen, Germany) in a 5:1 (v/v) ratio. The average mass deviation did not exceed 100 ppm. The MS experiments were conducted with the following settings: ion source 1, 25.09 kV; ion source 2, 23.80 kV; pulsed ion extraction, 260 ns, lens 6.40 kV, matrix suppression cut off m/z 700. For the acquisition and processing of the spectra, FlexControl 3.4 (Bruker Daltonics, Bremen, Germany) software was applied. ClinProTools 3.0 (Bruker Daltonics, Bremen, Germany) software was used for the chemometric analyses of the obtained data. Statistical analyses were performed with mathematical classification algorithms (quick classifier (QC), genetic algorithm (GA), supervised neural network (SNN)) and ROC curves. For each algorithm, parameters of cross-validation and recognition capability were calculated. In order to perform external validation, analyzed group of samples were divided into two subgroups. 62 samples (25 with T1DM and 27 healthy participants) were randomly selected as “learning” set, while 26 samples (15 with T1DM and 11 healthy participants) were randomly selected as “test” set. Correct classified part of valid spectra [%] was calculated. According to the obtained results, we depicted the peaks for the subsequent identification.

### nanoLC MALDI-TOF/TOF MS discriminatory peaks identification

Identification of the discriminative proteins and peptides was performed with nanoLC-MALDI-TOF/TOF MS (nano-liquid chromatography-matrix-assisted laser desorption/ionisation-time of flight/time of flight mass spectrometry) system. Serum samples obtained from patients were pretreated with ZipTip C18 micropipette tips and subjected to nanoLC separation. The nanoLC set consisted of EASY-nLC II (Bruker Daltonics, Bremen, Germany), nanoflow HPLC system, and Proteineer-fc II (Bruker Daltonics, Bremen, Germany) collector of fractions. The nano system parts were: NS-MP-10 BioSphere C18 (NanoSeparations, Nieuwkoop, the Netherlands) trap column (20 mm × 100 µm I.D., particle size 5 µm, pore size 120 Å), and an Acclaim PepMap 100 (Thermo Scientific, Sunnyvale, CA, USA) column (150 mm × 75 µm I.D., particle size 3 µm, pore size 100 Å). The gradient elution method was 2%-50% of ACN in 96 min (mobile phase A—0.05% TFA in water, mobile phase B—0.05% TFA in 90% ACN). For the separation, the flow rate was set at 300 nL/min, and the volume of the sample eluent injected into the chromatography column was 4 µL. From nanoLC separation, 384 separated fractions were obtained. Each fraction was mixed with a matrix solution. The matrix solution consisted of 36 µL of HCCA saturated solution in 0.1% TFA and acetonitrile (90:10 v/v), 748 µL of acetonitrile and 0.1% TFA (95:5 v/v) mixture, 8 µL of 10% TFA, and 8 µL of 100 mM ammonium phosphate. Such mixtures were spotted automatically onto the AnchorChip Standard (Bruker Daltonics, Bremen, Germany) MALDI target plate by the collector of fractions. HyStar 3.2 (Bruker Daltonics, Bremen, Germany) software was used for the nanoLC system operating. MS experiments were performed with UltrafleXtreme (Bruker Daltonics, Bremen, Germany) mass spectrometer working in a reflector mode in the range of m/z 700–3500. External calibration was performed with a mixture of Peptide Calibration Standard (Bruker Daltonics, Bremen, Germany). A list of the precursor ions for the identification was established with WARP-LC (Bruker Daltonics, Bremen, Germany) software. Parameters of MS and MS/MS mode were as follows: ion source 1, 7.50 kV; ion source 2, 6.75 kV; reflectron 1, 29.50 kV; reflectron 2, 14.00 kV; lens, 3.50 kV; lift 1, 19.00 kV; lift 2, 3.00 kV, pulsed ion extraction time, 80 ns. For the spectra collection, processing, and evaluation, FlexControl 3.4, FlexAnalysis 3.4 and, BioTools 3.2 (Bruker Daltonics, Bremen, Germany) software were used. For the identification of discriminative proteins and peptides, a SwissProt database and Mascot 2.4.1 search engine with taxonomic restriction to Homo sapiens were applied. The protein search parameters were set on: fragment ion mass tolerance m/z ± 0.7, precursor ion mass tolerance ± 50 ppm, peptide charge + 1, monoisotopic mass.

### Quantification of selected discriminative proteins by ELISA kit

ELISA kits for complement C3, complement C4A, and kininogen-1 (Cloud-Clone Corp., Houston, TX, USA) were used for the quantitative analysis of the selected proteomic features. Some of the discriminative m/z features corresponded to the fibrinogen alpha chain, but commercially available ELISA kits for this protein are not intended for the analysis of human serum. Therefore, we decided not to perform this analysis.

Tests were performed according to the manufacturers' protocols. Infinite M200PRO Microplate Reader (Tecan, Männedorf, Switzerland) and Magellan 7.1 software (Tecan, Männedorf, Switzerland) were used for acquiring of the results. Concentrations are presented in [ng/mL].

### Additional laboratory measurements in women with T1DM

Blood samples were taken after overnight fasting and immediately transported to the central laboratory of the Gynecologic Obstetrical University Hospital in Poznan for analysis. HbA1c level in whole blood was determined using the turbidimetric inhibition immunoassay (TINIA) (Tina-quant Hemoglobin A1c II test in a Cobas c311 analyser (Roche Diagnostics, Basel, Switzerland)).

The total serum cholesterol, HDL cholesterol and triglyceride (TG) levels were determined with Roche Diagnostics reagents (Cholesterol CHOD-PAP, HDL-C plus, and Triglycerides GPO-PAP, respectively) on a Cobas c501 analyser. The following formula was used to calculate the level of LDL cholesterol: LDL cholesterol = total cholesterol − HDL cholesterol − (TG/5).

### Statistical analysis

Statistical analysis was performed using MedCalc for Windows, version 19.8 (MedCalc Software, Mariakerke, Belgium). Testing for normality of data distribution was performed using the D'Agostino-Pearson test. Independent samples t-tests were used for groups' comparisons when data had normal distribution (data presented as means and standard deviations). When data were not normally distributed, Mann–Whitney tests were applied (data presented as medians and interquartile ranges).

The Spearman rank correlation coefficient (rho) was used to test the relationship between detected proteins' concentrations and clinical and laboratory data. Multiple regression (enter method) was applied to test for possible associations and interactions between chosen proteins' concentrations and clinical/laboratory parameters. Statistical significance was defined as p < 0.05 (two-sided).

### Institutional review board statement

The study was conducted according to the guidelines of the Declaration of Helsinki, and approved by the Institutional Review Board of Poznan University of Medical Sciences (no. 132/13 (7 February 2013); 223/13 (7 March 2013); 241/13 (7 March 2013); 194/14 (13 February 2014)).

### Informed consent statement

Informed consent was obtained from all subjects involved in the study.

## Results

### Demographic information

Women diagnosed with T1DM (29 ± 4 years old) in our study were significantly younger than controls (32 ± 4 years old, p = 0.005). Mean gestational week at blood collection differed among the groups, being significantly lower in women with T1DM (p < 0.0001) (Table 1). Newborns of women with T1DM were significantly heavier than those of controls. Both groups of women were comparable in terms of height, weight at the beginning of the pregnancy, and at term, BMI, and gestational weight gain. There were no cases of serious adverse perinatal outcomes in the study groups (fetal malformations, intrauterine fetal deaths, preeclampsia, fetal growth restriction). Characteristics of the study groups are shown in Table 1.

**Table 1:** Characteristics of the study groups.

	T1DM	Controls	p-value
Number of patients (n)	40	38	–
Age, years (SD)	29 (4)	32 (4)	0.005
Duration of diabetes, years (SD)	13 (7)	–	–
Age at time of diagnosis of DM, years (IQR)	13 (10–24)	–	–
Proliferative retinopathy + overt nephropathy (n)	1	–	–
Proliferative retinopathy (n)	6	–	–
Peripheral/autonomic neuropathy (n)	3	–	–
Patients height, cm (SD)	167 (7)	167 (5)	0.93
Patients weight at term, kg (IQR)	75 (72–84)	74 (68–83)	0.29
Patients weight at the beginning of pregnancy, kg (IQR)	63.2 (58.5–69.5)	61.0 (56.0–68.0)	0.18
BMI at the beginning of pregnancy, kg/m^2^ (IQR)	22 (21–26)	22 (20–23)	0.13
Gestational weight gain, kg (SD)	13.7 (4.6)	14.1 (4.4)	0.88
Blood collection, gestational week (SD)	38 (1)	39 (1)	< 0.0001
Media HbA1c I trimester, % (IQR)	6.4 (5.9–7.2)	–	–
Median HbA1c II trimester, % (IQR)	5.6 (5.3–6.0)	–	–
Median HbA1c III trimester, % (IQR)	5.8 (5.5–6.2)	–	–
Newborns’ birthweights, g (SD)	3591 (536)	3350 (357)	0.02

### MALDI-TOF protein-peptide profiling

**Figure 1:**
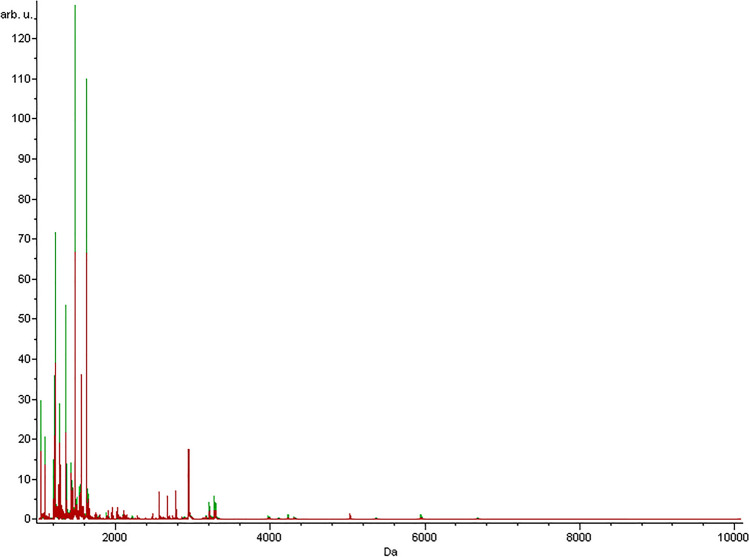
Average MALDI-TOF MS spectra of serum samples acquired for studied groups. Spectra of patients diagnosed with T1DM (red) and controls (green) are presented over the full scan range of m/z 1000–10,000.

**Figure 2:**
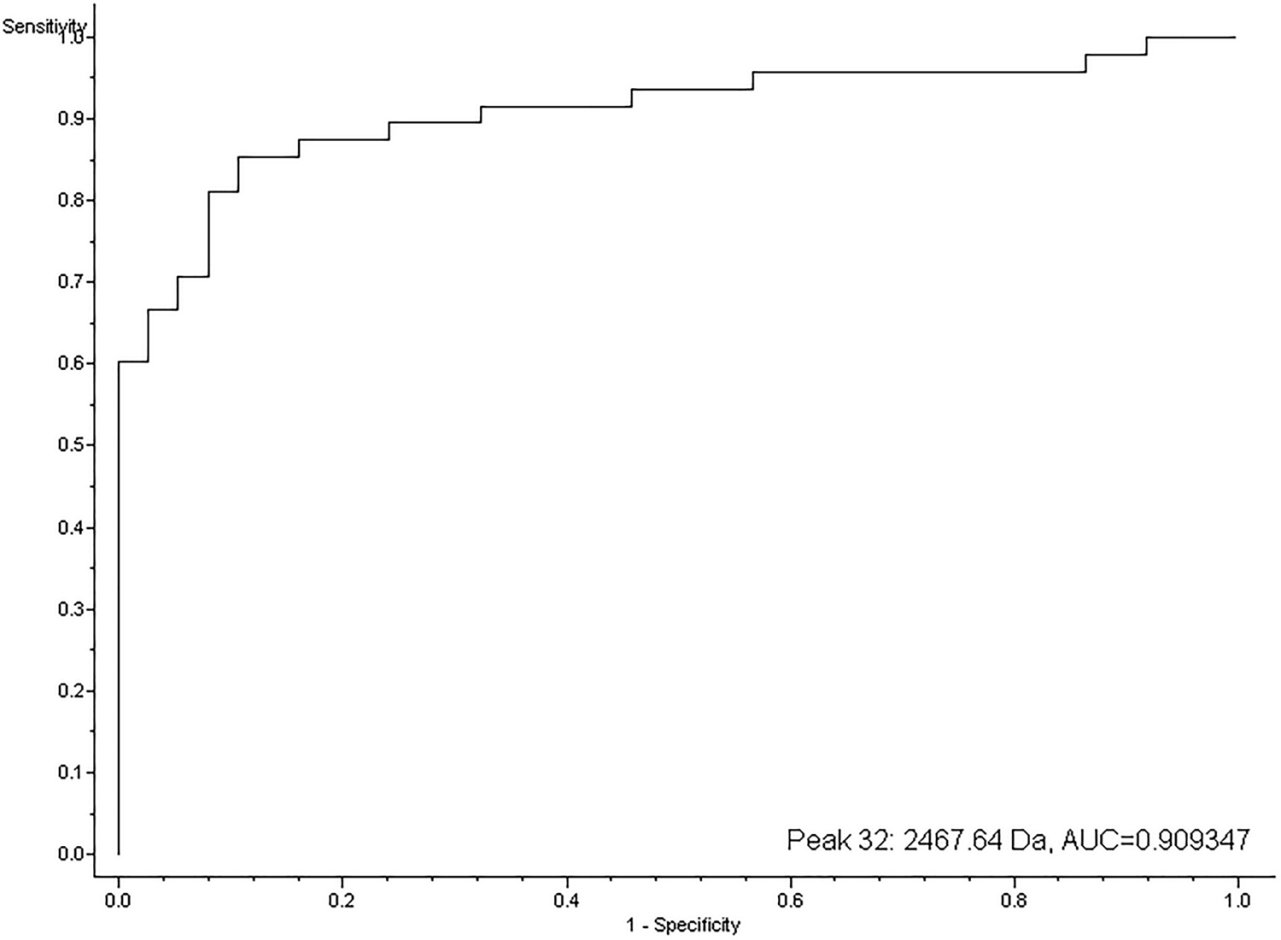
Receiver operating characteristic (ROC) curve representing sensitivity and specificity of peak of m/z 2467.64. Area under the ROC curve (AUC) is 0.91.

Total average spectra of the test and control groups are presented in Fig. 1. For the analysis of the proteomic data obtained from MALDI-TOF MS analyses, ROC curves and area under ROC curves were calculated, and three different chemometric algorithms (GA, SNN, QC) were applied (Fig. 2). The algorithms are based on different mechanisms. Therefore peaks classified as discriminative between groups are divergent^13^. Lists of discriminative m/z ions for each algorithm are presented in Table 2. Chemometric methods used in the study were also discussed in detail elsewhere^14^.

**Table 2:** Peaks classified as discriminative using genetic algorithm (GA), supervised neural network (SNN) and quick classifier (QC).

GA	SNN	QC
1865.35	5934.84	1189.14
1740.48	1740.48	1276.92
3996.61	2973.50	2467.64
1189.14	4220.01	2847.05
1487.94	3304.46	5934.84
2934.66	2770.84	
3293.64	2210.01	
6672.97	2956.26	
4220.01	1419.13	
1435.04	2661.38	
1519.27	1616.83	
5359.15	1261.30	
1220.43	2081.79	
1021.92	1545.76	
1276.92	2946.55	
	3234.45	
	1896.65	
	1638.77	

For each algorithm, the values of cross-validation, external validation, and recognition capability were calculated. The cross-validation value determines the reliability of the algorithm-based model. It is a method allowing for evaluating a classifier's performance using certain parameters based on specific data. It works by automatically assigning data to two sets: a "learning" set and a "test" set. The "learning" set, using the selected classifier, determines the model. The "test" set is used to evaluate the obtained model. It also allows to estimate the forecasting ability. This process is repeated many times, leading to the determination of the relative predictive capability, calculated based on accumulated absolute values of the predictive power. Cross-validation of the results obtained in this analysis was performed using the "Leave One Out" method. It is based on excluding one of the obtained results (the remaining ones constitute a "learning" set) and then using it as a "test" set. The choice of this method was dictated by the size of the test and control groups. In this study, the highest cross-validation value was calculated for GA; it was 93.4% (Table 2). Recognition capability is expressed by the percentage of individuals correctly assigned to the studied groups. The highest recognition capability value (97.9%) was calculated for both GA and SNN algorithms. External validation in principle is similar to cross-validation but requires additional spectra (obtained from both the test and control groups) that were not previously included in the set used in "learning" the model. These "new" spectra are classified based on of the previously obtained model. In this study, external validation was performed using independent data set (pregnant women with type 1 diabetes n = 15 and healthy pregnant controls n = 11). The highest values of external validation parameters were obtained for the GA-based model (Table 3).

**Table 3:** Values of chemometric parameters calculated for algorithms (GA—genetic algorithm; SNN—supervised neural network; QC—quick classifier).

Model	Cross-validation [%]	Recognition capability [%]	External validation—correct classified part of valid spectra [%]	
			Test	Control
GA	93.4	97.9	97.7	100.0
SNN	92.7	97.9	86.4	90.9
QC	86.1	88.2	70.5	100.0

### nanoLC-MALDI-TOF/TOF MS/MS identification of the discriminative peaks

Application of MALDI-TOF/TOF tandem mass spectrometry allowed for the identification of four proteins based on the discriminative m/z features. Fibrinogen alpha chain was identified according to the peptide fragments of m/z 1189.14; 1021.92; 2661.38; 1616.83; and 2467.64 (Table 4). Within them, the peak of m/z 1189.14 was classified as discriminative for both GA and QC. Precursor ions of m/z 1740.48; 1435.04; 1896.65 were identified as fragments of complement C4-A (peak of m/z 1740.48 was classified as discriminative in both GA and SNN). Complement C3 was identified based on two peaks depicted as differentiating for the GA model: m/z 1865.35; and 1519.27. The last identified precursor ion was kininogen-1, according to the mass of m/z 2081.79, classified as discriminative for SNN.

**Table 4:** Identified proteins classified as discriminative between studied groups.

Model	m/z values	P-value of Wilcoxon test	Fragmentation sequence	Identified proteins
GA	1865.35	0.0000238	R.SSKITHRIHWESASLL.R	Complement C3
	1740.48	0.202	R.NGFKSHALQLNNRQI.R	Complement C4-A
	1189.14	< 0.000001	G.EGDFLAEGGGVR.G	Fibrinogen alpha chain (Glu→pyro-Glu: 1)
	1435.04	0.116	R.GLEEELQFSLGSK.I	Complement C4-A
	1519.27	0.0000303	G.SPMYSIITPNILR.L	Complement C3 (oxidation)
	1021.92	0.000517	G.DFLAEGGGVR.G	Fibrinogen alpha chain
SNN	1740.48	0.202	R.NGFKSHALQLNNRQI.R	Complement C4-A
	2661.38	0.742	A.DEAGSEADHEGTHSTKRGHAKSRPV.R	Fibrinogen alpha chain
	1616.83	0.00000362	T.ADSGEGDFLAEGGGVR.G	Fibrinogen alpha chain
	2081.79	0.573	K.HNLGHGHKHERDQGHGHQ.R	Kininogen-1
	1896.65	0.519	R.NGFKSHALQLNNRQIR.G	Complement C4-A
QC	1189.14	< 0.000001	G.EGDFLAEGGGVR.G	Fibrinogen alpha chain (Glu→pyro-Glu: 1)
	2467.64	< 0.000001	S.SSYSKQFTSSTSYNRGDSTFES.K	Fibrinogen alpha chain

The MALDI-TOF/TOF MS/MS analyses were performed in the mass range of m/z 700–3500. Above the upper limit, the resolution significantly decreases. Hence, peaks of m/z 3996.61; 6672.97; 4220.01; 5359.15; and 5934.84 could not be identified. Moreover, some precursor ions below m/z 3500 could not be detected, probably due to the presence of neighbouring peaks. These precursors require further identification.

### Quantification of selected discriminative proteins by ELISA kits

The serum concentration of kininogen-1 was significantly lower in women with T1DM than in controls. There were no significant differences in serum concentrations of complement C3 and complement C4-A between study groups. The detailed results are shown in Table 5.

**Table 5:** Serum concentrations of selected proteins (ELISA).

	T1DM	CONTROLS	p-value
Kininogen-1, ng/mL (SD)	924.5 (392.6)	1151.2 (252.2)	0.003
Complement C3, µg/mL (IQR)	719.2 (665.9–775.2)	736.4 (665–803)	0.43
Complement C4-A, µg/mL (IQR)	57.3 (44.9–81.1)	60.3 (48.3–112.9)	0.27

Kininogen concentration was significantly correlated with maternal age in controls-rho = 0.383; p = 0.0177. Such correlation was not observed in women with T1DM. Importantly, in multiple regression analysis in the whole study group (T1DM and controls), maternal age was not associated with kininogen concentration. The only determinant of kininogen-1 concentration was diagnosed T1DM (regression coefficient = 258.9; p = 0.0016; R2-adjusted = 0.1).

There were no correlations between kininogen/complement C3/complement C4-A and pregestational BMI/gestational weight gain in both women with diabetes and controls. Neither was there correlation between levels of kininogen/complement 3/complement C4-and T1DM duration.

A significant positive correlation was observed between complement C4-A and triglycerides measured before delivery in women with T1DM- rho = 0.48; p = 0.0034. Similarly, a significant positive correlation was observed between complement C3 and LDL measured before delivery in women with T1DM—rho = 0.345; p = 0.049. Kininogen/complement C3/complement C4-A were not correlated with maternal HbA1c in all three trimesters in women with type 1 diabetes. No significant correlations were observed between kininogen/complement C3/complement C4-A and newborns’ birthweights in both groups.

## Discussion

To the best of our knowledge, this is the first study analyzing the differences of serum peptide profiles among healthy pregnant patients and those with T1DM. Peptides and proteins that differentiate the two groups can be considered potential markers of T1DM in late pregnancy. Using MALDI-TOF MS, and nano-MALDI-TOF/TOF MS, we have identified kininogen-1, complement C3, and C4-A as potential indicators for T1DM in pregnancy. Consequently, validation with ELISA was utilized to quantify identified proteins. Kininogen-1 was found to be less abundant in sera of pregnant women with T1DM, while no significant difference in serum concentration was observed for complement C3 and C4-A between the two groups.

Kininogen-1, also referred to as high molecular weight kininogen (HMWK), was identified as a peptide peak m/z 2081.79. This protein has been classified as discriminative using the SNN algorithm. The chemometric analyses were performed on a semi-qualitative MALDI dataset dedicated to the method. Moreover, additional analyses were performed using the ELISA technique to obtain complete quantitative data. Kininogen-1 was less abundant in sera of pregnant women with T1DM. Since the difference in kininogen concentrations was confirmed by the qualitative ELISA test, this protein may be considered a potential indicator of T1DM state. Kininogen-1 seems to be the most important result obtained in this study. Kininogen is believed to participate in the initiation of blood coagulation cascade, complement system activation, and is closely linked to the renin-angiotensin system via the angiotensin-converting enzyme (ACE). Kininogen is converted into a small peptide, kinin, by the enzyme called kallikrein. Kinins, specifically, are believed to be potent renal vasodilators with concomitant antithrombotic and antifibrotic functions. In the context of T1DM, kinins are believed to serve a protective role against the development of microalbuminuria and eventually diabetic nephropathy^15^. An experimental study in mice has correlated kallikrein deficiency with the development of microalbuminuria. Similar results were observed with high ACE enzyme levels. ACE is known to convert angiotensin I into angiotensin II, however, it also plays a role of the kinin-degrading enzyme, resulting in significant degradation of kinin while having only a limited impact on angiotensin II production. Additionally, ACE I/D polymorphism has been previously associated with the development of diabetic nephropathy, while the ACE II genotype is believed to be nephroprotective in T1DM and T2DM^16^. In fact, previous proteomic studies have associated differential expression of kininogen-1 with the evolution of microalbuminuria, thus allowing it to serve as an early marker of nephropathy associated with T1DM and T2DM^15,17–19^.

Vitova et al.^19^ suggest an association between microalbuminuria in patients with T1DM and decreased activity of the kallikrein system. Specifically, they identified diminished urinary excretion of kininogen -1 heavy chain in T1DM non-pregnant patients with microalbuminuria compared to those without microalbuminuria. It was concluded that decreased activity of the kallikrein system, including kininogen, locally or systemically, was associated with the development of microalbuminuria. Since our study was meant to explore the potential serum biomarkers specific to T1DM in pregnancy, urine samples were not collected for proteomics. However, there were no women with diabetic nephropathy in our study group. If decreased systemic activity of the kallikrein system, is associated with development of diabetic nephropathy and associated complications, diminished serum kininogen-1 identified in our study may be an early marker of kidney function deterioration in pregnant women with T1DM not yet seen in routine urinalysis. Another study has also identified kininogen-1, as well as mannan-binding lectin serine protease 2, and prothrombin, as potential biomarkers for microalbuminuria in an attempt to prevent and diagnose diabetic nephropathy in T2DM patients at an early stage^17^. Similar to the study by Vitova et al.^19^ decreased urinary excretion of four identified biomarkers, including kininogen-1, was evident in patients with T2DM with microalbuminuria compared to those without microalbuminuria and healthy controls. Since all four identified biomarkers are believed to play a role in the complement cascade, decreased excretion was concluded to indicate dysfunction in immune response^17^. Since both studies have evaluated urine proteomic profiles alone, while our study analyzed serum protein composition, the significance of the relationship between the two profiles, as well as the utility of either method for clinical evaluation of diabetic nephropathy, requires further examination. Controversially, a previous study in rats with T1DM have reported upregulation of kininogen levels in urine^20^.

Another study utilized proteomic analysis of plasma samples to establish a correlation with early progressive renal function decline in macroalbuminuric patients with T1DM^21^. Unlike in the current study, the mean abundance of kininogen-1 (three fragments) and a fragment of plasma kallikrein-sensitive glycoprotein (inter-alpha-trypsin inhibitor heavy chain H4, ITIH4) were increased by 30–50% in T1DM patients who were at risk of early progressive renal function decline, compared to those with normal renal function. Additionally, proteomic profiling in rats with induced T1DM revealed increased serum expression of kininogen in the aorta and the kidneys^22^. Interestingly, this effect was believed to be modulated by hyperglycemia since treatment with insulin and control of blood glucose levels reversed the expression of kininogen. Although our study has identified decreased levels of kininogen-1 in serum, such discrepancy in result may be attributed to the animal model, level of blood glucose during the blood draw, or overall blood glucose control.

In the context of our result, decreased kininogen-1 in pregnant women T1DM may indicate a higher risk of developing diabetic nephropathy with microalbuminuria, which is associated with maternal and fetal complications. Diabetic nephropathy in pregnant women with T1DM was associated with a higher prevalence of preeclampsia (48%) and pre-term delivery (73%), compared to pregnant T1DM without diabetic nephropathy (preeclampsia—24%, pre-term delivery 44%)^23^. Also, intrauterine growth restriction was twice more common in pregnant women with T1DM and diabetic nephropathy compared to those with normal kidney function. However, due to a minimal number of women with nephropathy and a lack of pregnancy data on microalbuminuria, we could not draw reliable conclusions on the possible impact of proteomic changes and kidney function in our cohort.

Interestingly, kininogen-1 (along with lumican) have been identified as potential biomarkers for late and early pre-term birth due to their differential expression in amniotic fluid samples^24^. Wen et al.^25^ have identified kininogen-1 as one of the 19 serum peptides that could serve as a predictor of preeclampsia (PE) or be used in the differential diagnosis of PE from confounding chronic hypertension^4,25^. Our study group consisted of uncomplicated women who continued pregnancy up to term, however, it would be reasonable to design a new proteomic study in complicated diabetic pregnancies. If kininogen-1's utility as a biomarker is confirmed, it might be incorporated in routine screening in T1DM during pregnancy to assess the risk for development of diabetic nephropathy, associated complications including pre-term delivery and preeclampsia, or to develop early management strategies for such patients.

In the current study, complement C4-A and C3 were identified in serum based on fragments of m/z 1740.48; 1435.04; 1896.65 and 1865.35; 1519.27, respectively. However, unlike in the case of kininogen-1, there was no significant difference in their serum concentrations between the two study groups on validation using ELISA. The main aim of protein-peptide profiling is to compare the whole profiles established from MS spectrum data. Mathematical algorithms allow obtaining models based on the most characteristic features. However, the presence of a particular peptide in the created model does not always indicate that the whole protein would be dysregulated in the study group. Therefore, the additional validation of the obtained results is necessary. Classically, complement protein C3 is believed to play a role in the activation of the lecithin complement pathway, however, there is also evidence of its implication in insulin resistance. Studies of C3-deficient mice, however, have indicated decreased insulin level and improved glucose tolerance^26^. Plasma levels of C3 mRNA in adipose tissue have also been negatively correlated with insulin sensitivity^27^. Notably, serum complement C3 was shown to have a stronger association with insulin resistance than highly sensitive C-reactive protein in non-diabetic Chinese patients^28–30^. In recent years, insulin resistance has been implicated in the pathogenesis of T1DM in pregnancy and may predispose patients to miscarriage, preeclampsia, and macrosomia^31^. Downregulation of complement protein C3 was also observed in studies with T2DM patients^18,32,33^. In studies of pregnant women with GDM, both maternal C3-A and C4-A concentrations were significantly lower compared to healthy women at the time of delivery^34^. With that said, no significant difference in cord plasma levels of C3-A, C4-A and C5A was observed in women from both groups of the aforementioned study. However, women with GDM included in this study had a relatively high BMI (28.1 ± 1.1 kg/m^2^ at 12 weeks of gestation; 31.3 ± 1.1 kg/m^2^ at delivery) compared to women with T1DM in our study (Table 1). In fact, recently published study reports increased levels of C3 and C4 during the second trimester of pregnancy in women diagnosed with GDM to be independently associated with their disease and, rather to be attributed to the level of inflammation and high BMI^35^. Additionally, level of CRP in plasma, a reliable indication of inflammation, was a predictor of C3 and C4 elevation. Levels of C3 and C4 were no longer significantly elevated when regression model accounted for CRP, ALT and systolic blood pressure. Another study has noted a downregulation of C4-A in post-mortem testing of patients with sudden infant death syndrome^36^.

On the contrary, analysis of vitreous humor in patients with diabetic nephropathy identified upregulation of complement protein C3 (along with apolipoprotein A1, APOH, fibrinogen, C4b, C9 and factor B)^37^. Another study identified higher concentrations of glycated complement C4-A in patients with T1DM^38^.

In the another study, amniotic fluid analysis of 15 women with preeclampsia and healthy controls did not reveal differences in C4A between the two groups^39^. However, different study highlights the implication of C4A and apolipoprotein A-1 plasma level measurement in distinguishing women based on the onset and severity of preeclampsia^40^. Significantly lower plasma concentrations of C4A were observed in women with severe, early-onset preeclampsia compared to those with severe, late-onset preeclampsia. Based on the current studies, there is no consensus about the roles of C4A and C3 in diabetes or pregnancy-associated complication. Based on the previous studies, the significance of C4 serum levels in T1DM during pregnancy remains unclear, and further investigations are required.

Identification of unique biomarkers in a setting of T1DM in pregnancy may be useful in early diagnosis and prediction of the risk of maternal and fetal complications as a result of disease progression. According to previous studies, kininogen-1 has some utility in predicting microalbuminuria and diabetic nephropathy in patients with T1DM and T2DM, however, its utility as a biomarker in T1DM disease progression in pregnancy requires further clinical evaluation. Although this study was able to address it’s main goal in establishing the proteomic fingerprint of T1DM in pregnancy, a future study should focus on evaluating levels of identified biomarkers in pregnant patients with T1DM that develop diabetes specific complications (such as microalbuminuria and diabetic nephropathy).

Proteome-wide profiling still remains to be a powerful tool in up-to-date science, however it is not free of limitations that have been addressed in previous projects^41,42^. Our previous studies confirmed, that this approach is accurate for characterization and identification of proteomic patterns of different diseases^14,43^. During the analysis, the m/z range of 1–10 kDa was examined, therefore, it could be assumed that only peptide fraction was analyzed. Peptide fraction in blood mainly occurs as the effect of natural proteolysis and depends on the activity and specificity of proteolytic enzymes, enzymatic stability of the particular peptide, and many others. Since, there is no certainty that obtained peptide pattern reflects protein composition, the immunoenzymatic tests (or other quantitative analysis) are necessary. However, the main aim of the profiling is to establish the specific fingerprint, characteristic for the study health condition, not an identification of a single marker. The additional identification of the differentiating m/z, may only suggest that the concentrations of the identified proteins are changed under the influence of the disease. The obtained results, which strongly suggest differences in the peptide composition, may reflect the occurrence of some changes in the process of proteolysis or in the proteins structures (see Table 4). Differential expression of proteases as well as protease inhibitors, has already been associated with other diseases, like breast cancer^44^.

The major limitation of this study was that due to a minimal number of complications observed in women with type 1 diabetes in this cohort, we could not establish links between proteomic changes and these complications. However, this study lays the grounds for further proteomic studies in this field.

## Conclusions

Our study showed a specific proteomic profile in women with T1DM compared to those without the disease. While this study highlights major differences in proteins that participate in coagulation and inflammatory pathways, their utility as biomarkers of T1DM-associated pregnancy complications require further investigation.

## Data Availability

The datasets generated during and/or analyzed during the current study are available from the corresponding author on reasonable request.
